# Early versus late supplemental parenteral nutrition in gastrointestinal cancer patients undergoing major abdominal surgery: 5-year outcomes from a randomized clinical trial

**DOI:** 10.1097/JS9.0000000000004504

**Published:** 2026-03-12

**Authors:** Xuejin Gao, Yupeng Zhang, Yaqin Xiao, Tingting Gao, Gang Jin, Kunhua Wang, Yanbing Zhou, Qiang Chi, Hua Yang, Mengbin Li, Jianchun Yu, Huanlong Qin, Yun Tang, Xiaoting Wu, Guoli Li, Li Zhang, Xinying Wang

**Affiliations:** a Department of General Surgery, Jinling Hospital, Affiliated Hospital of Medical School, Nanjing University, Nanjing, China; b Department of General Surgery, Jinling Hospital, Nanjing Medical University, Nanjing, China; c Department of General Surgery, Jinling Hospital, School of Medicine Southeast University, Nanjing, China; d Department of Hepatobiliary Pancreatic Surgery, Changhai Hospital, The Second Military Medical University, Shanghai, China; e Department of General Surgery, First Affiliated Hospital of Kunming Medical University, Kunming, China; f Department of Gastrointestinal Surgery, The Affiliated Hospital of Qingdao University, Qingdao, China; g Department of General Surgery, The Second Affiliated Hospital of Harbin Medical University, Haerbin, China; h Department of General Surgery, The Second Affiliated Hospital, Army Medical University, Chongqing, China; i Department of Gastrointestinal Surgery, The First Affiliated Hospital of Air Force Medical University, Xi’an, China; j Department of General Surgery, Peking Union Medical College Hospital, Chinese Academy of Medical Sciences, Beijing, China; k Department of General Surgery, Shanghai Tenth People’s Hospital, School of Medicine, Tongji University, Shanghai, China; l Department of General Surgery, First Medical Center of Chinese PLA General Hospital, Beijing, China; m Department of Gastrointestinal Surgery, West China Hospital, Sichuan University, Chengdu, China

**Keywords:** 5-year overall survival, gastrointestinal cancer, major abdominal surgery, supplemental parenteral nutrition

## Abstract

**Background:**

The long-term effects of supplemental parenteral nutrition (SPN) on survival and recurrence in gastrointestinal (GI) cancer patients undergoing major abdominal surgery remain unclear. This study reports the 5-year overall survival (OS) results from the PNASIT randomized clinical trial (RCT), which compared early SPN [initiated on postoperative day (POD) 3] versus late SPN (initiated on POD 8) in patients with GI undergoing major abdominal surgery.

**Methods:**

This multicenter, open-label, RCT was conducted at 11 centers in China. From 1 April 2017 to 31 December 2018, a total of 217 eligible patients with nutritional risk (Nutritional Risk Screening 2002) score ≥3) who were undergoing elective major abdominal surgery for GI cancer were enrolled. Patients were randomized into two groups: the early SPN group (E-SPN; *n* = 107) and the late SPN group (L-SPN; *n* = 110). The primary endpoint was 5-year OS, analyzed using Kaplan–Meier methods and Cox proportional hazards models on an intention-to-treat basis.

**Results:**

Among the 217 analyzed patients [mean (SD) age, 60.4 (12.2) years; 60.4% male; all of Asian ethnicity], the 5-year OS rate was 70.1% (75/107) in the E-SPN group and 66.4% (73/110) in the L-SPN group [hazard ratio (HR), 0.857; 95% confidence interval (CI), 0.535–1.374; log-rank *P* = 0.520]. No significant differences were observed in 5-year disease-free survival rates, recurrence rates, or recurrence patterns between the two groups (all *P* > 0.05). HRQoL, as measured by the 36-Item Short Form Health Survey scale, was significantly higher in the E-SPN group compared with the L-SPN group during the first 6 postoperative months (*P* < 0.001). However, no significant differences were observed at 1, 3, or 5 years after surgery.

**Conclusion:**

In GI cancer patients undergoing major abdominal surgery, early SPN showed no significant difference in 5-year OS compared with late SPN. The findings do not support the use of early SPN to improve long-term survival in this patient population.

**Trial registration:**

ClinicalTrials.gov Identifier: NCT03115957.

## Introduction

The reported prevalence of malnutrition in patients with cancer ranges from approximately 20% to 70% worldwide, with variations depending on patient age, cancer type, and cancer stage[1]. The underlying causes of nutritional impairment include tumor-mediated catabolism, inflammation, mechanical obstruction, and gastrointestinal (GI) dysfunction due either to the malignancy itself or its treatment^[2,3]^. The clinical consequences of malnutrition are profound and extend beyond nutritional deficiency, exerting systemic effects. Numerous studies have established malnutrition as an independent risk factor for adverse postoperative outcomes, including increased morbidity, prolonged hospital stays, higher mortality, reduced efficacy of adjuvant therapies, and impaired quality of life^[4–6]^. It is estimated that malnutrition contributes to 10–20% of cancer-related deaths, independent of the malignancy itself[1].

Given the profound impact of malnutrition on clinical outcomes, nutritional support has become a critical component of multimodal cancer care[1]. According to ESPEN and ASPEN guidelines, enteral nutrition (EN) is recommended as the primary route of feeding^[7,8]^. The perioperative period is increasingly recognized as a critical window for nutritional intervention. Modern clinical nutrition strategies have evolved from basic energy supplementation to incorporate immunonutrition, which aims to enhance immune cell function, reduce inflammation and postoperative complications, and modulate immune responses^[9,10]^. However, achieving full nutritional requirements through EN alone in the early postoperative period is often impractical. Therefore, supplemental parenteral nutrition (SPN) is recommended when EN provides less than 50% of energy requirements, particularly during the perioperative period^[7,11]^. The optimal timing for initiating SPN in the context of contemporary multimodal care remains an area requiring further investigation. The PNASIT trial (early vs delayed parenteral nutrition in abdominal surgical patients) demonstrated that early initiation of SPN (POD 3) significantly reduced nosocomial infection rates compared with late SPN (POD 8) in patients undergoing major abdominal surgery[12]. Despite these short-term benefits – such as reduced nosocomial infections and improved nutritional status^[12–15]^ – the long-term impact of early SPN on 5-year survival and cancer recurrence remains unclear in GI cancer populations.

Therefore, we conducted this preplanned follow-up study of the PNASIT randomized clinical trial (RCT) to evaluate the effect of early versus late SPN on 5-year survival rates in GI cancer patients undergoing major abdominal surgery.

This manuscript adheres to the TITAN 2025 guidelines for transparent reporting of artificial intelligence use in scientific writing, and the completed TITAN Guideline Checklist 2025 is submitted as supplementary material[16].

## Methods

### Study design

We conducted a preplanned analysis of long-term follow-up data from the PNASIT trial. The PNASIT trial was an investigator-initiated, multicenter, open-label, RCT conducted in the general surgery departments of 11 academic hospitals in China. The trial protocol and the statistical analysis plan are available in Supplemental Digital Content 2, available at: http://links.lww.com/JS9/H50. The institutional review boards at all participating sites approved the trial protocols, and the study was conducted in accordance with the Declaration of Helsinki. All participants provided written informed consent prior to enrollment. Both the original trial and this secondary analysis adhere to the Consolidated Standards of Reporting Trials (CONSORT) guideline[17].

HIGHLIGHTS

- In gastrointestinal cancer patients undergoing major abdominal surgery, compared with late SPN, early SPN did not improve 5-year overall survival (70.1% vs. 66.4%; HR, 0.857; 95% CI, 0.535–1.374; *P* = 0.520).
- There were no significant differences in 5-year disease-free survival rates, overall recurrence rates, or recurrence patterns between the groups.
- Health-related quality of life (SF-36 scale) was significantly higher in the early SPN group during the first 6 postoperative months (*P* < 0.001), but this difference disappeared at 1, 3, and 5 years after surgery.

### Study population

Using prospectively collected long-term follow-up data from the PNASIT trial, we focused on the GI cancer cohort per the predefined objectives. This resulted in 217 eligible patients, who were randomized to either the early SPN (E-SPN; *n* = 107) or late SPN (L-SPN; *n* = 110) group.

The PNASIT trial enrolled adults (18–80 years) undergoing major nontraumatic abdominal surgery who had a Nutritional Risk Screening 2002 (NRS 2002) score ≥3, an expected postoperative stay >7 days, and achieved ≤30% of energy targets via EN by POD 2[12]. Based on these criteria, 230 patients were randomly allocated in a 1:1 ratio to either the early SPN group (initiating SPN on POD 3 and achieving 100% of defined energy targets) or the late SPN group (initiating SPN on POD 8 and achieving 100% of defined energy targets). Energy targets were defined as 30 and 25 kcal/kg of ideal body weight for men and women, respectively. The detailed inclusion and exclusion criteria have been previously published[12] and are also provided in Supplemental Digital Content eAppendix 1, available at: Supplemental Digital Content 1, available at: http://links.lww.com/JS9/H51.

The assigned nutritional intervention (early vs late SPN) was confined to the initial postoperative hospitalization. Adherence to the protocol-specified initiation day (POD 3 for E-SPN, and POD 8 for L-SPN) and energy targets was monitored by the study investigators. After discharge, all patients received standard institutional nutritional counseling and support, regardless of their initial randomization group.

### Data collection and baseline measurements

Baseline patient characteristics – including sex, age, weight, body mass index (BMI), NRS-2002 score, disease diagnosis, tumor type, surgical procedure, comorbidities, Eastern Cooperative Oncology Group (ECOG) performance status, pathological tumor-node-metastasis (TNM) stage, use of adjuvant chemotherapy, type of operation, duration of surgery, blood loss, and blood transfusion – were collected.

### Outcomes

The primary endpoint of the study was 5-year overall survival (OS). Secondary endpoints included 5-year disease-free survival (DFS), recurrence patterns, and health-related quality of life (HRQoL), which was assessed using the 36-Item Short Form Health Survey (SF-36) evaluating both physical and mental component summaries. OS was calculated from the time of randomization until either the occurrence of death (event) or the last follow-up examination (censored).

Patients were followed for a minimum of 60 months after surgery. The follow-up protocol included (1) comprehensive clinical evaluation, including medical history and physical examination; (2) measurement of tumor markers (CEA, AFP, and CA19-9) for monitoring disease progression; (3) radiological surveillance with chest and abdominal CT scans; (4) endoscopic evaluation of the GI tract. The surveillance interval was individualized according to tumor-specific characteristics and established guidelines. Positron emission tomography–computed tomography was performed when recurrence was suspected. Recurrence was identified through a combination of medical history, physical examination, imaging evaluation, cytology, or tissue biopsy (preferred when feasible)[18].

### Statistical analysis

The primary outcome, 5-year OS, was analyzed on an intention-to-treat basis, as were all secondary outcomes including DFS, recurrence, and HRQoL. To minimize missing data on survival and recurrence events, comprehensive follow-up protocols were implemented using hospital records, telephone interviews, and national databases. HRQoL was assessed using the SF-36 at predefined intervals (postoperative months 1, 3, 6, 12, 36, and 60). During extended follow-up, missing HRQoL data arose from loss to follow-up, withdrawal of consent, disease progression or death, and incomplete questionnaires. To address missing data, the following procedure was implemented: first, descriptive comparisons of baseline characteristics were conducted between patients with complete and missing HRQoL data to evaluate potential patterns of missingness. Subsequently, multiple imputation methods were applied to handle the missing HRQoL values.

Demographic characteristics were summarized using descriptive statistics. Categorical variables were expressed as frequencies and percentages, and continuous variables were presented as means with standard deviations or medians with interquartile ranges, based on their distribution. Normality of data was assessed using the Shapiro–Wilk test. Between-group comparisons were performed using the Chi-square test or Fisher’s exact tests for categorical variables, and independent *t*-tests or Mann–Whitney *U* tests for continuous variables, as appropriate based on distributional assumptions. Survival analysis was conducted using the Kaplan–Meier method to compare 5-year OS between groups. We used Cox proportional hazards regression to assess the effects of age, sex, type of operation, adjuvant chemotherapy, TNM stage, duration of surgery, and blood loss on survival time. We also performed competing risk regression to account for non-primary cancer-related deaths and used the Fine–Gray test to compare primary cancer-specific cumulative incidence between the E-SPN and L-SPN groups.

Data were analyzed using SPSS, version 25 (IBM Corp), and SAS software, version 9.4 (SAS Institute Inc). All *P* values were two sided, and statistical significance was set at *P* < 0.05. Analyses were performed between January 1 and 2 March 2025.

## Results

### Study participants

**Figure 1.**
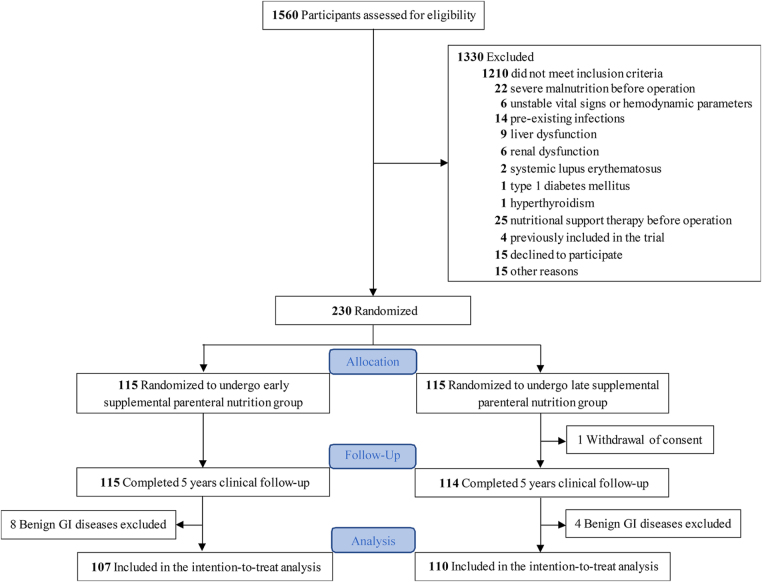
CONSORT flow diagram of study participant enrollment and randomization.

Between 1 April 2017 and 28 February 2019, a total of 2048 patients were screened. Of these, 230 eligible patients were enrolled in the PNASIT trial. After excluding 12 patients with benign GI diseases (8 in the E-SPN group and 4 in the L-SPN group), the remaining 217 patients were randomized into two groups: the early SPN group (E-SPN, n = 107) and the late SPN group (L-SPN, n = 110) (Fig. 1).

The study included 217 patients. The mean (SD) age was 60.4 (12.2) years; 131 patients (60.4%) were men and 86 (39.6%) were women, and all of Asian ethnicity. The median follow-up period was 52 months (IQR, 36–62 months). Most patients were diagnosed with TNM II stage or higher; however, 41 patients (18.9%) had TNM I stage, including 20 (18.7%) in the E-SPN group and 21 (19.1%) in the L-SPN group. Adjuvant chemotherapy was administered to 56 patients (52.3%) in the E-SPN group and 58 (52.7%) in the L-SPN group, with no significant difference between the groups. The other baseline characteristics were also similar in the two groups (Table 1).

**Table 1:** Baseline demographic and clinical characteristics.

Characteristic	E-SPN group (*n* = 107)	L-SPN group (*n* = 110)	*P*-value
Sex, *n* (%)			0.869
Female	43 (40.2)	43 (39.1)	
Male	64 (59.8)	67 (60.9)	
Age (years)	60.6 ± 12.8	60.2 ± 11.8	0.811
Height (cm)	164.6 ± 8.3	164.8 ± 8.5	0.861
Weight (kg)	62.0 ± 11.3	62.7 ± 11.2	0.647
BMI (kg/m^2^)	22.9 ± 3.3	23.1 ± 3.4	0.661
NRS-2002 score, *n* (%)^a^			
3 points	86 (80.4)	88 (80.0)	0.945
≥4 points	21 (19.6)	22 (20.0)	
Diagnosis, *n* (%)			
Gastric cancer	39 (36.4)	36 (32.7)	0.344
Colorectal cancer	40 (37.4)	46 (41.8)	
Pancreatic cancer	12 (11.2)	17 (15.5)	
Cholangiocarcinoma	1 (0.9)	3 (2.7)	
Other GI cancers^b^	15 (14.0)	8 (7.3)	
Comorbidity, *n* (%)			0.897
Comorbidities^c^	13 (12.1)	14 (12.7)	
ECOG performance status, *n* (%)			0.970
0	80 (74.8)	82 (74.5)	
1	27 (25.2)	28 (25.5)	
Pathological TNM stage, *n* (%)			0.999
I	20 (18.7)	21 (19.1)	
II	40 (37.4)	42 (38.2)	
III	44 (41.1)	44 (40.0)	
IV	3 (2.8)	3 (2.7)	
Received chemotherapy			0.954
Yes	56 (52.3)	58 (52.7)	
No	51 (47.7)	52 (47.3)	
The operation type, *n* (%)			0.845
Laparotomy	52 (48.6)	52 (47.3)	
Laparoscopic surgery	55 (51.4)	58 (52.7)	
Surgical procedure, *n* (%)			0.332
Gastrectomy (total/subtotal)	43 (40.2)	40 (36.4)	
Colectomy/Rectectomy	40 (37.4)	46 (41.8)	
PD/PPPD	13 (12.1)	17 (15.5)	
Major hepatectomy	1 (0.9)	3 (2.7)	
Others	10 (9.3)	4 (3.6)	
Duration of surgery, *n* (%)			0.776
<2 h	10 (9.3)	13 (11.8)	
2–5 h	83 (77.6)	81 (73.6)	
>5 h	14 (13.1)	16 (14.5)	
Blood loss, *n* (%)			0.775
≤500 mL	94 (87.9)	98 (89.1)	
>500 mL	13 (12.1)	12 (10.9)	
Blood transfusion, *n* (%)	14 (13.1)	12 (10.9)	0.622

E-SPN, early supplemental parenteral nutrition; L-SPN, late supplemental parenteral nutrition; BMI, body mass index; NRS 2002, Nutritional Risk Screening 2002; ECOG, Eastern Cooperative Oncology Group; GI, gastrointestinal.

Data are number of participants (%) or mean (SD) unless otherwise noted. Continuous data described as mean (SD) were compared using the *t*-test or Mann–Whitney *U* test. Outcomes expressed as percentages of patients with each outcome were compared between the two groups using the χ^2^ or Fisher’s exact test.

^a^ Scores on nutritional risk screening 2002 range from 0 to 7, with a score of 3 or more identifying patients at nutritional risk. Higher scores indicate increased risk.

^b^ Indicates small intestinal tumor, gastrointestinal stromal tumor.

^c^ Comorbidities included type 2 diabetes mellitus, arthritis, and hypertensive diseases.

### Primary outcome: OS

**Figure 2.**
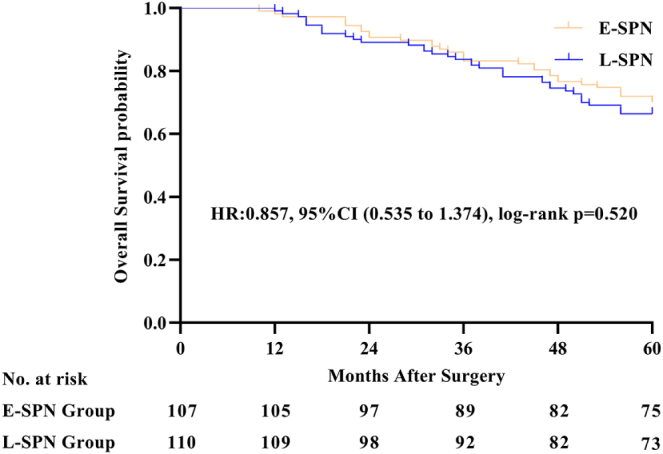
Overall survival for early versus late supplemental parenteral nutrition during the 5-years’ follow-up.

At the final follow-up in the intention-to-treat analysis, 69 patients (31.8%) had died, including 32 in the E-SPN group and 37 in the L-SPN group. The 5-year S rate was 70.1% (75/107) in the E-SPN group and 66.4% (73/110) in the L-SPN group [hazard ratio (HR), 0.857; 95% confidence interval (CI), 0.535–1.374; log-rank *P* = 0.520] (Fig. 2 and eTable 1 in Supplemental Digital Content 1, available at: http://links.lww.com/JS9/H51). The HR for E-SPN vs L-SPN group was 0.871 (95% CI, 0.546–1.416; *P* = 0.551) after we adjusted for age, sex, BMI, ECOG performance status, and comorbidity (eTable 1 in Supplemental Digital Content 1, available at: http://links.lww.com/JS9/H51). Univariate analysis of 5-year OS showed no significant differences between the E-SPN and L-SPN groups in terms of age, sex, type of operation, adjuvant chemotherapy, TNM stage, duration of surgery, or blood loss (eTable 2 in Supplemental Digital Content 1, available at: http://links.lww.com/JS9/H51). Causes of death were analyzed for all patients, with the distributions presented in Table 2. In a competing risks analysis (Fine−Gray model), the subdistribution hazard ratio [sHR] for death from primary cancer did not differ significantly between groups (sHR, 0.885; 95% CI, 0.529–1.481; *P* = 0.642), treating deaths from other causes as a competing event.

**Table 2:** Frequencies of Causes of First Recurrence and Death Within 5 Years.

Events	E-SPN Group (n = 107)	L-SPN Group (n = 110)	Between-group difference (95% CI)	*P*-value
5-year DFS	69 (64.5)	68 (61.8)	2.67 (−6.71 to 12.05)	0.684
Any recurrence^a^				
Local	14 (13.1)	16 (14.5)	−1.46 (−8.17 to 5.25)	0.755
Peritoneum	8 (7.5)	10 (9.1)	−1.61 (−6.97 to 3.74)	0.666
Liver	10 (9.3)	11 (10.0)	−0.65 (−6.40 to 5.09)	0.871
Multiple sites^b^	3 (2.8)	2 (1.8)	0.98 (−1.94 to 3.91)	0.680
Other or uncertain sites^c^	3 (2.8)	3 (2.7)	0.08 (−3.11 to 3.27)	0.973
Cause of death	32 (29.9)	37 (33.6)	−3.73 (−12.78 to 5.32)	0.555
Primary cancer	27 (25.2)	31 (28.2)	−2.95 (−11.55 to 5.65)	0.624
Other causes^d^	5 (4.7)	6 (5.5)	−0.78 (−5.04 to 3.48)	0.793

E-SPN, early supplemental parenteral nutrition; L-SPN, late supplemental parenteral nutrition; CI, confidence interval; DFS, disease-free survival.

^a^ Refers only to first-time recurrence, even though patients can have recurrence at multiple times.

^b^ Includes patients who have recurrence simultaneously in two or more metastatic sites, including peritoneum, liver, lung, bone, brain, distant lymph node, or other hematogenous metastatic sites.

^c^ Includes hematogenous recurrence at sites other than liver (ie, lung, bone, brain), recurrence at distant lymph node, and recurrence at uncertain sites.

^d^ Includes other cancers, diseases other than cancer, unintentional injuries, and unknown causes.

### Secondary outcomes: DFS, recurrence, and HRQoL

In the intention-to-treat analysis, the 5-year DFS rate − calculated based on time-to-event analysis of patients who died or experienced recurrence − was 64.5% (69/107) in the E-SPN group and 61.8% (68/110) in the L-SPN group (risk difference 2.67%; 97.5% CI [−6.71 to 12.05]; *P* = 0.684) (Table 2). Recurrence was observed in 38 patients (cumulative incidence: 35.5%) and in 42 patients (cumulative incidence: 38.2%) in the E-SPN and L-SPN groups, respectively. The cumulative incidence of specific recurrence types did not differ significantly between the groups (all *P* > 0.05) (Table 2). The sites of recurrence were also similar between the 2 groups (*P* > 0.05) (Table 2). Furthermore, there was no significant difference in the cumulative incidence of recurrence or death between the groups (HR, 0.893; 95% CI, 0.576–1.384; log-rank *P* = 0.609) (eFigure 1 in Supplemental Digital Content 1, available at: http://links.lww.com/JS9/H51).

**Figure 3.**
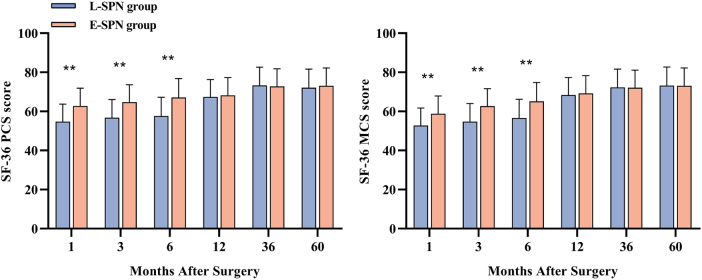
Health-related quality of life with early vs late supplemental parenteral nutrition during the 5-years’ follow-up.**P < 0.001.

HRQoL, assessed using the SF-36, was significantly higher in the early SPN group compared with the late SPN group during the first 6 months postoperatively (including both physical and mental component summaries; *P* < 0.001) (Fig. 3 and eTable 3 in Supplemental Digital Content 1, available at: http://links.lww.com/JS9/H51). However, this improvement diminished over time, and no statistically significant differences in HRQoL were observed between the groups at 1, 3, or 5 years after surgery (Fig. 3 and eTable 3 in Supplemental Digital Content 1, available at: http://links.lww.com/JS9/H51).

## Discussion

This pre-planned 5-year follow-up of the PNASIT trial provides the first multicenter randomized controlled trial evidence comparing long-term outcomes between early and late SPN in GI cancer patients undergoing major abdominal surgery. The findings demonstrated no statistically significant differences between the early and late SPN groups with respect to 5-year OS (70.1% vs. 66.4%; HR, 0.857; 95% CI, 0.535–1.374; *P* = 0.520), DFS, recurrence rates, or recurrence patterns. Although early SPN significantly improved HRQoL (SF-36 scores) during the first 6 months postoperatively, this benefit was not sustained at 1, 3, or 5 years. The findings do not support the use of early SPN to improve long-term survival in this patient population but provide new evidence to guide the optimization of clinical nutritional support strategies.

Our study provides critical evidence for updating long-term guidance in nutritional support. Current ESPEN/ASPEN guidelines recommend SPN for its established short-term benefits − a rationale confirmed by our previous findings of fewer nosocomial infections and improved nutritional status[12]. However, guidelines are silent on long-term survival. Therefore, our study provides the first high-level evidence that these short-term advantages do not translate into improved 5-year survival in GI cancer surgery patients. International guidelines recommend SPN to meet energy and protein requirements in patients with cancer when EN alone is insufficient^[7,8,11]^. Previous studies have demonstrated that SPN can improve nutritional status, enhance postoperative recovery, and strengthen immune function by delivering essential nutrients that bolster host defenses against infections.^[19,21]^ Our study demonstrates that early SPN did not significantly improve OS in patients with GI cancer, suggesting that although it may facilitate recovery and short-term improvements in HRQoL, it does not alter the underlying biology of tumor progression or metastatic dissemination. Although early SPN could theoretically exert indirect effects on tumor prognosis by rapidly improving postoperative nutritional status, reducing inflammation, and enhancing immune function^[13,14,21]^, long-term follow-up data indicate that such short-term advantage does not translate into a long-term survival. Possible explanations for these findings include the following: (1) this study included patients with various GI cancers (e.g., gastric, colorectal, and pancreatic), each with distinct biological characteristics and prognoses. As survival in oncology is predominantly determined by factors such as tumor type, TNM stage, and molecular characteristics, this heterogeneity may have diluted any potential survival advantage of early SPN. (2) The dual-edged nature of SPN: while early initiation may improve nutritional status, it might also facilitate tumor progression through overfeeding, liver injury, and metabolic disturbance. (3) Differences in survival outcomes may be obscured by other therapeutic factors, such as the standardization of adjuvant chemotherapy or the use of targeted therapies.

The findings of the present study are consistent with the results of previous studies of early PN in critically ill patients[22] and preoperative PN in patients with gastric cancer on long-term survival[23]. As shown in earlier study, early SPN did not improve 2-year survival or physical functioning in critically ill adults, even among those at the highest nutritional risk, suggesting that early SPN has no significant effect long-term survival and functional outcomes[22]. Similarly, previous studies have indicated that short-term preoperative PN does not significantly improve long-term survival in patients with gastric cancer after surgery[23], which aligns with our study that also focused on GI cancer patients evaluating the effect of PN on survival. However, our results contrast with clinical trials in palliative care settings for advanced cancer, where PN was associated with improved survival in patients with malignant bowel obstruction^[24,25]^. This discrepancy may be attributed to differences in patient populations (e.g., disease severity, cancer type, and clinical context) and intervention duration. It should be noted that, although appropriate perioperative nutritional support therapy can reduce the risk of postoperative complications in patients undergoing high-risk GI surgery^[26,28]^, its long-term effect on cancer recurrence or metastasis remains uncertain. Our study finding aligns with previous large trials in critical care and surgical oncology, where appropriate nutritional support therapy has consistently improved short-term outcomes but failed to enhance long-term survival. This recurring pattern of transient benefits in recovery without ultimate survival impact underscores that while early SPN is a valuable supportive measure, it does not alter the underlying determinants of cancer prognosis.

Our study demonstrated that the timing of standard SPN alone is not a decisive factor for long-term survival. This insight compels a paradigm shift from a one-size-fits-all approach toward precision nutrition. Future strategies must be personalized, moving beyond universal timing to incorporate the patient’s inflammatory status, metabolic phenotype, and body composition. Consequently, the pivotal question evolves from “when to feed” to “whom to feed, and with what,” based on their specific physiological state. This conceptual pivot is supported by extant evidence. The survival benefit of early SPN is known to be less pronounced in patients without severe malnutrition, and its potential can be diminished by metabolic complications like hyperglycemia and liver injury due to overfeeding^[29,30]^. These findings are highly consistent with the emerging personalized nutrition therapy or precision nutrition therapy in clinical practice^[31,33]^. Therefore, future nutritional intervention strategies should incorporate baseline metabolic status, inflammatory markers, and dynamic metabolic responses to nutrients. This approach would help establish individualized “metabolic readiness” for nutrition support and enable dynamic, timely adjustments in protein, energy, and micronutrient supplementation to maximize clinical outcomes.

Perioperative nutrition in GI oncology is increasingly integrated into multidisciplinary care, a model proven to improve survival, quality of life, and overall treatment success[34]. Although our study primarily investigated the timing of nutritional support, nutritional composition may be equally critical. Previous study suggests that perioperative immunonutrition can improve clinical outcomes in GI cancer patients[10]. Therefore, our study provides a pivotal insight: within modern protocols, merely advancing the timing of SPN does not alter long-term oncology outcomes. This underscores the imperative to reorient the focus from a universal timing strategy toward a personalized, multimodal clinical nutrition strategy.

Our study showed a transient improvement in functional status, as measured by SF-36 PCS scores in the first 6 months, which did not extend to sustained benefits or long-term survival. The dissociation between transient HRQoL improvement and absent long-term survival benefit reveals that early SPN supports postoperative recovery without altering cancer’s fundamental drivers. This is explained by two key factors. First, per the “seed and soil” hypothesis, any transient improvement in the systemic “soil” is insufficient to durably suppress micrometastatic seeds. Second, standard SPN lacks the immunomodulating nutrients necessary to reprogram antitumor immunity for long-term control. Consequently, the dominant influence of tumor biology overrides short-term metabolic support, meaning early SPN aids recovery without changing the cancer’s course.

### Limitations

Although this multicenter RCT provides valuable insights into the long-term effects of SPN, several limitations should be acknowledged. First, it is important to note that the original PNASIT trial was primarily designed with nosocomial infection as the primary endpoint. Therefore, the present analysis of 5-year OS should be interpreted as an important exploratory assessment of long-term outcomes, rather than a definitive hypothesis-testing analysis. Although the randomized design ensures balanced baseline characteristics, the sample size was not sufficiently powered to detect small but potentially clinically meaningful differences in survival outcomes. Second, the inclusion of patients with diverse GI cancer types constitutes a fundamental limitation of this study. This heterogeneity may have masked any potential benefit of nutritional timing, as tumor type and stage are known to substantially influence survival outcomes. Although this design enhances the generalizability of our findings, it correspondingly reduces the statistical power for detecting subgroup-specific effects. This view is supported by a previous large-scale study conducted in Japan, which confirmed that factors such as TNM stage and surgical approach significantly impact long-term prognosis in GI cancer[35]. Therefore, future research should focus on conducting tumor-specific analyses to better evaluate the effects of nutritional interventions in more homogeneous patient populations. Third, although major confounders were controlled for, the potential influence of unmeasured factors, including tumor molecular characteristics and variations in adjuvant treatment, cannot be ruled out. Furthermore, our trial was not designed to conduct subgroup analyses by specific tumor type or stage. Consequently, future studies should focus on enrolling large, homogeneous cohorts to definitively assess whether specific patient subgroups (e.g., those with advanced gastric cancer or distinct metabolic phenotypes) derive long-term benefit from early SPN. Fourth, despite the use of standardized protocols, individual variations in nutrient metabolism and microbiome-mediated nutrient utilization may have modulated treatment effects; these factors were not measured. Fifth, our nutritional status assessment relied on conventional biomarkers (albumin and prealbumin), which have inherent limitations. These parameters are influenced by nonnutritional factors (e.g., interleukin-6 and C-reactive protein) and thus may not accurately reflect true nutritional status. This limitation is particularly relevant given the increasing emphasis on more comprehensive nutritional and metabolic profiling (e.g., GLIM criteria, sarcopenia, inflammatory indices) in contemporary surgical oncology. Finally, the generalizability of our findings may be limited by the single-country design and the concentration of care in specialized academic centers with high-level expertise. These limitations collectively underscore the need for future research to move beyond timing alone and focus on personalized nutritional strategies, taking into account individual metabolic profiles, inflammatory status, and tumor biology, in order to identify subgroups of patients who might genuinely benefit from specific nutritional interventions.

## Conclusion

This secondary analysis of long-term follow-up data from a previously published open-label RCT demonstrates that early SPN resulted in similar 5-year OS compared with late SPN in patients undergoing major abdominal surgery for GI cancer. The findings do not support the use of early SPN to improve long-term survival in this patient population.

## Data Availability

If interested in the study data and individual-patient data, please contact the corresponding author and the data will be shared with bona fide researchers with research proposals after research completion.

## Research registration unique identifying number (UIN)

The study was registered at ClinicalTrials.gov (identifier: NCT03115957).
